# *Lysinibacillus spp*.: an IAA-producing endospore forming-bacteria that promotes plant growth

**DOI:** 10.1007/s10482-023-01828-x

**Published:** 2023-05-03

**Authors:** Manuel Pantoja-Guerra, Marleny Burkett-Cadena, Johanna Cadena, Christopher A. Dunlap, Camilo A. Ramírez

**Affiliations:** 1 Universidad de Antioquia, Instituto de Biología, Medellín, Colombia; 2 Plant Response Biotech, Plant City, FL USA; 3 United States Department of Agriculture, Agricultural Research Service, National Center for Agricultural Utilization Research, Crop Bioprotection Research Unit, 1815 N University, Peoria, IL USA; 4 Facultad de Ciencias Agropecuarias, Unilasallista Corporación Universitaria, Caldas - Antioquia, Colombia

**Keywords:** *Lysinibacillus*, PGPR, Indole-3-acetic acid, Bio-stimulation

## Abstract

*Lysinibacillus* is a bacterial genus that has generated recent interest for its biotechnological potential in agriculture. Strains belonging to this group are recognized for their mosquitocidal and bioremediation activity. However, in recent years some reports indicate its importance as plant growth promoting rhizobacteria (PGPR). This research sought to provide evidence of the PGP activity of *Lysinibacillus spp*. and the role of the indole-3-acetic acid (IAA) production associated with this activity. Twelve *Lysinibacillus spp*. strains were evaluated under greenhouse conditions, six of which increased the biomass and root architecture of corn plants. In most cases, growth stimulation was evident at 10^8^ CFU/mL inoculum concentration. All strains produced IAA with high variation between them (20–70 µg/mL). The bioinformatic identification of predicted genes associated with IAA production allowed the detection of the indole pyruvic acid pathway to synthesize IAA in all strains; additionally, genes for a tryptamine pathway were detected in two strains. Extracellular filtrates from all strain’s cultures increased the corn coleoptile length in an IAA-similar concentration pattern, which demonstrates the filtrates had an auxin-like effect on plant tissue. Five of the six strains that previously showed PGPR activity in corn also promoted the growth of *Arabidopsis thaliana* (col 0). These strains induced changes in root architecture of *Arabidopsis* mutant plants (aux1-7/axr4-2), the partial reversion of mutant phenotype indicated the role of IAA on plant growth. This work provided solid evidence of the association of *Lysinibacillus spp*. IAA production with their PGP activity, which constitutes a new approach for this genus. These elements contribute to the biotechnological exploration of this bacterial genus for agricultural biotechnology.

**Supplementary Information:**

The online version contains supplementary material available at 10.1007/s10482-023-01828-x.

## Introduction

Biofertilizers and biostimulants are emerging as alternatives for sustainable agricultural management (Adesemoye and Kloepper 2009; Mahanty et al. 2017; Rouphael and Colla 2020). PGPR (Plant Growth Promoting Rhizobacteria) is the microbial group with the best potential to serve as agricultural bio-inputs. Many successful experiences of PGPR-based products have been reported which have led to a decrease in the use of chemical fertilizers in several agricultural systems (Ghosh 2004; Yao et al. 2006; Kennedy 2008; Naveed et al. 2015). With short generation times, the rapid bacterial biomass production in vitro is ideal to produce biological inoculants at an industrial scale.

The genus *Lysinibacillus* is a group of rod-shaped endospore-forming bacilli. Their main phenotypical distinctive trait is the content of Lys-D-Asp residues in the peptidoglycan of their cell wall (Ahmed et al. 2007). Additionally, their respiratory lipoquinone system is made up of Menaquinone MK-7, and the most abundant polar lipids in its cell membrane are formed by diphosphatidylglycerol, phosphatidylglycerol, and ninhydrin-positive phosphoglycolipids (Aimé et al. 2019).

Recently, *Lysinibacillus spp*. have gained interest for biotechnological applications (Hernández-Santana et al. 2022). Strains belonging to *L. sphaericus *sensu lato have insecticide properties against mosquitoes from the genera *Culex*, *Anopheles*, *Mansonia*, and *Aedes sp*. (Aimé et al. 2019). This activity is mediated by several toxins: a parasporal binary toxin (Bin-toxin) (Berry 2012; Glare et al. 2017); a family of vegetative toxins (Mtx 1–4) (Berry 2012; Allievi et al. 2014); and sphaericolysin, a toxin insecticide against *Blattela germanica* and *Spodoptera litura*. The toxin complex Cry48/Cry49 also has been described and have shown activity against *Culex* larvae (Berry 2012).

The importance of *Lysinibacillus sp*. in bioremediation processes, both for heavy metals and organic pollutants, has also been studied. Bahuguna et al. (2011) described the dibenzothiophene desulfurization by *Lysinibacillus sphaericus* DMT-7 isolated from diesel contaminated soil. Gupta et al. (2012) reported the sequestration of 97% of mercury chloride in the *L. fusiformis* biomass, where the mercury was transformed into mercuric chloride and 63% of it was volatilized. *Lysinibacillus sp*. KMKA was able to degrade orange M2R dye and simultaneously reduce CrVI (Chaudhari et al. 2013). *L. sphaericus* B1-CDA can accumulate up to 5 mg of arsenic by gram of bacterial biomass in a liquid medium (Rahman et al. 2014). *Lysinibacillus fusiformis* G2, isolated from an oil-contaminated soil, can produce biosurfactant substances with emulsifying properties on hydrocarbons (Kumar et al. 2015). *L. boronitolerans* IICT-akl252 has the able to degrade nitriles.

Likewise, *Lysinibacillus sp.* are valuable plant growth promoters and can offer protection to agriculturally important crops. Vendan et al. (2010) isolated *L. fusiformis* EI20 as an endophytic bacteria associated with ginseng plants. This strain presented several PGPR traits including a high indole-3-acetic acid (IAA) production. *L. fusiformis* B-CM18 was isolated from the chickpea rhizosphere and showed several PGPR and biocontrol traits (Singh et al. 2013). *Lysinibacillus* sp. EB45, EB53, and EB50 showed PGPR activity in banana plants (Andrade et al. 2014). *L. fusiformis* Lf89 induced the root growth and proliferation of *Arabidopsis* and *Datura* plants (Rahmoune et al. 2017). The inoculation of *Lysinibacillus sphaericus* ZA9 on cucumber and tomato increased the germination percentage, the vigor of plants, and height of seedlings. This strain displayed a high IAA production that could be associated with the PGPR activity (Naureen et al. 2017). Burkett-Cadena et al. (2019) reported the isolation of *Lysinibacillus capsici* PB300, which presented PGPR activity with great potential for biofertilizer design. This background suggests that the *Lysinibacillus* genus has potential in agricultural biotechnology.

In this research, we evaluated the PGP activity of 12 *Lysinibacillus spp*. strains on corn, and we found evidence of its association with bacterial IAA production. First, the PGPR activity of *Lysinibacillus spp.* strains on a model of agricultural interest (maize) was evaluated. Then, the role of IAA bacterial production in this activity was evaluated. To associate the PGP activity of *Lysinibacillus spp.* to an auxin effect, several evidence lines were generated: IAA bacterial production in vitro, genetic association, coleoptile elongation test with free cell culture, and assays with auxin mutant *Arabidopsis* plants.

## Materials and methods

### Strains, growth conditions, and cell suspension preparation

Four *Lysinibacillus spp*. strains were supplied by Pathway-Biologic® (currently Plant Response Inc.) (PB211, PB293, PB300, and PB512), and were isolated from agricultural soils during bioprospecting processes. The eight types strains were supplied by the United States Department of Agriculture—USDA (CBP678, CBP1140, CBP1002, CBP1617, CBP1619, CBP1621, CBP1622, CBP1624). All twelve strains were frozen at − 80 °C until use. Reactivation was done on Trypticase Soy Agar (TSA) plates incubated at 28 °C. Bacterial suspensions of 10^8^, 10^6^, and 10^4^ CFU/mL were made in sterile distilled water from all strains to perform the experiments.

### Phylogenomic analysis of *Lysinibacillus spp*.

For the three strains that did not have publicly available genomes (PB211, PB293 and PB512), they were cultured in TSA to early stationary phase (~ 24 h) and then harvested. DNA extraction was performed on the bacterial biomass using a DNeasy Ultraclean Microbial kit (Qiagen Inc, Cambridge, MA). The genomic DNA was prepared for sequencing using Nextera XT library preparation kit following the manufacturer’s suggested protocols. The prepared libraries were sequenced using a MiSeq DNA sequencer using the MiSeq V3 2 × 300 sequencing kit. The resulting reads were quality trimmed to the Q30 confidence level. The draft genome was assembled using CLCbio Genomics Workbench 11.0 (Qiagen Inc, Cambridge, MA) using default parameters. The genomes were accession into GenBank as PB211 (JANTOO01), PB293 (JANTON01) and PB512 (JANTOM01).

Genome alignments for the phylogenetic tree were made using BIGSdb software (Jolley and Maiden 2010). The phylogenetic tree was constructed using MEGA 7.0.26 software (Kumar et al. 2016). Neighbor-joining trees were reconstructed using the Tamura-Nei model (Tamura and Nei 1993) with a gamma correction (alpha value = 0.47); this model was chosen based on the likelihood test implemented in MEGA 7.0.26. Measures of bootstrap support for internal branches were obtained from 1,500 pseudoreplicates. A dataset of 1185 genes identified as the core genome of the *Lysinibacillus* type strains was found using BIGSdb software (Jolley and Maiden 2010) and used as the basis of the phylogenetic analysis.

### Evaluation of *Lysinibacillus spp*. PGP (plant growth promotion) activity on corn

Corn seeds (*Zea mays* ICA-109) were obtained commercially and were disinfected with 70% ethanol, then rinsed with sterile water. The seeds were air-dried on absorbent paper. The dried seeds were pregerminated on water-agar at 28 °C for 72 h. Seedlings with radicles of 1–2 cm long were selected for the experiment.

The seedlings were immersed for 1 h in sterile Petri dishes with different bacterial suspensions with inoculum concentrations of 10^4^, 10^6^, and 10^8^ CFU/mL, and sterile distilled water was used as a control treatment. The seedlings were sown under greenhouse conditions in PVC propagators (25 cm high × 8 cm diameter) and distributed using a completely random design. The propagators contained river sand moistened at 70% of its maximum moisture retention capacity. Samples were irrigated with 50% Hoagland nutrient solution and left to grow for two weeks. Each treatment consisted of 7 replicates and the experiment was performed twice. The data was analyzed by time blocks.

The response variables evaluated were shoot, root, and total dry weight, the root architecture variables were the number of main roots, total length of main roots, and the number of lateral roots. Root architecture parameters were obtained using digital image analysis with the Smart-Root package (Lobet et al. 2011, 2015).

### Lysinibacillus spp. IAA bacterial production

The strains were cultivated in TSA and incubated at 28 °C for 48 h, single colonies of each isolate were transferred to a 100 mL flask with 20 mL of Trypticase Soy Broth (TSB) supplemented with 500 µg/mL tryptophan and shaken at room temperature for an additional 48 h.

After culturing, the content of the flasks was placed in 50 mL falcon tubes, centrifuged at 3250 rpm for 15 min, and supernatant was collected. A mixture was prepared using 200 µL of supernatant and 800 µL of Salkowski indicator solution (ratio 1:4). The mixture was stirred and left standing for 20 min until the pink color stabilized. The absorbance was measured in a spectrophotometer at 535 nm. The concentration of IAA in the supernatant was estimated by interpolating the absorbance results on a standard curve with the concentrations of known IAA. The trial consisted of 5 replicates.

### Bioinformatic identification of predicted genes associated with *Lysinibacillus spp*. IAA production

The genomes of the twelve strains were analyzed for the presence of gene related to auxin biosynthesis pathways. Genome were annotated using the RAST server (Overbeek et al. 2014) imported and refined using Geneious Prime version 2019.1 (http://www.geneious.com, Kearse et al. 2012).

Based on the summary list related to auxin biosynthesis pathways, genes involved in each step were extracted from the genome using genomic annotations and the blast tool in Geneious 2019.1. Determination of unique/common characteristics among the 12 genomes was determined by blasting (≥ 70% homology) each respective annotated genome against a reference strain (one strain compared against 11 strains successively). With the information obtained from the list of genes, auxin biosynthetic pathways were assigned.

### Auxin-like activity of *Lysinibacillus spp*. filtrates: coleoptile elongation test

Corn ICA-109 seeds were pregerminated for 96 h on water-agar at room temperature and in dark conditions. After coleoptiles grew 1–2 cm, 5 mm segments were cut and placed in sterile distilled water while stirring at 150 rpm for 30 min. The segments were transferred to Petri dishes with 8 mL of IAA solutions at different concentrations (0 ppm [control—sterile distilled water], 1, 2.5, 5, 7.5, and 10 ppm). They were stirred at 150 rpm for 24 h at room temperature. The length was measured by image digital analysis using the ImageJ package (Abràmoff et al. 2004).

The method described by Idris et al. (2004) was used with modifications to evaluate the filtrates’ auxin-like effect of liquid bacterial culture. The strains were cultured in the same medium and conditions described above to evaluate the IAA bacterial production. The IAA concentration in the filtrates was brought to the same concentrations of IAA solutions previously evaluated in the coleoptile elongation test and 5 mm coleoptile segments were deposited in Petri dishes with 8 mL of the solutions prepared from the filtrates and stirred at 150 rpm for 24 h at room temperature. The length was measured by digital image analysis using the ImageJ package.

The cell suspensions’ auxin-like effect of each of the evaluated strain was also assessed. Coleoptile segments were deposited in 8 mL of suspensions with concentrations of 10^4^, 10^6^, 10^8^, and control (sterile distilled water). They were stirred at 150 rpm for 48 h and the length of the segments was evaluated with digital image analysis using the ImageJ package.

### In vitro experiments with wild and mutant plants: *Arabidopsis thaliana*

Wild type (col 0) and mutant (*aux1-7/axr4-2*) plants of *Arabidopsis thaliana* (Pickett et al. 1990; Hobbie and Estelle 1995; Puga-Freitas et al. 2012; Blouin 2018) were grown on Petri dishes with Murashige and Skoog (MS) medium (Sigma® M5519) in the presence of *Lysinibacillus spp*. Seeds were disinfected with a 30% NaClO solution from a 2.5% stock solution for 5 min. The seeds then were immersed in 70% ethanol for 5 min and rinsed 5 times with sterile distilled water.

The seeds were distributed in Petri dishes with MS medium using a micropipette tip, following the method proposed by Rivero et al. (2014) and placed in refrigeration (4 °C) for 48 h. The seeds were then pregerminated in a growth chamber Percival® E36-HO (25 °C; photoperiod, 12 h; light intensity, 1700 µmoles/m^2^/s) for two weeks. After pre-germination, three seedlings were transferred to Petri dishes with MS medium. The 6 strains previously showing PGP activity (PB211, PB293, PB300, PB512, CBP1622, and CBP1624) were inoculated 3.4 cm from the plants. The dishes were incubated by two weeks in growth chambers under the conditions previously described. Each treatment was performed in triplicate. As response variables, the plant fresh weights and root architecture variables were measured using the SmartRoot package (Lobet et al. 2011, 2015).

### Statistical analysis

All data were analyzed through linear models with analysis of variance (ANOVA). Significant differences in treatments regarding the controls were detected using the Dunnett test, and *P* values were reported in all cases. To detect differences between all treatments, the Tukey test was performed if necessary (alpha = 0.05). Data that could not be adjusted linearly by transformations were analyzed with nonparametric tests (Kruskal–Wallis and Wilcoxon). All data was analyzed using the R statistical package.

## Results and discussion

### Phylogenomic analysis of *Lysinibacillus spp*.

**Fig. 1:**
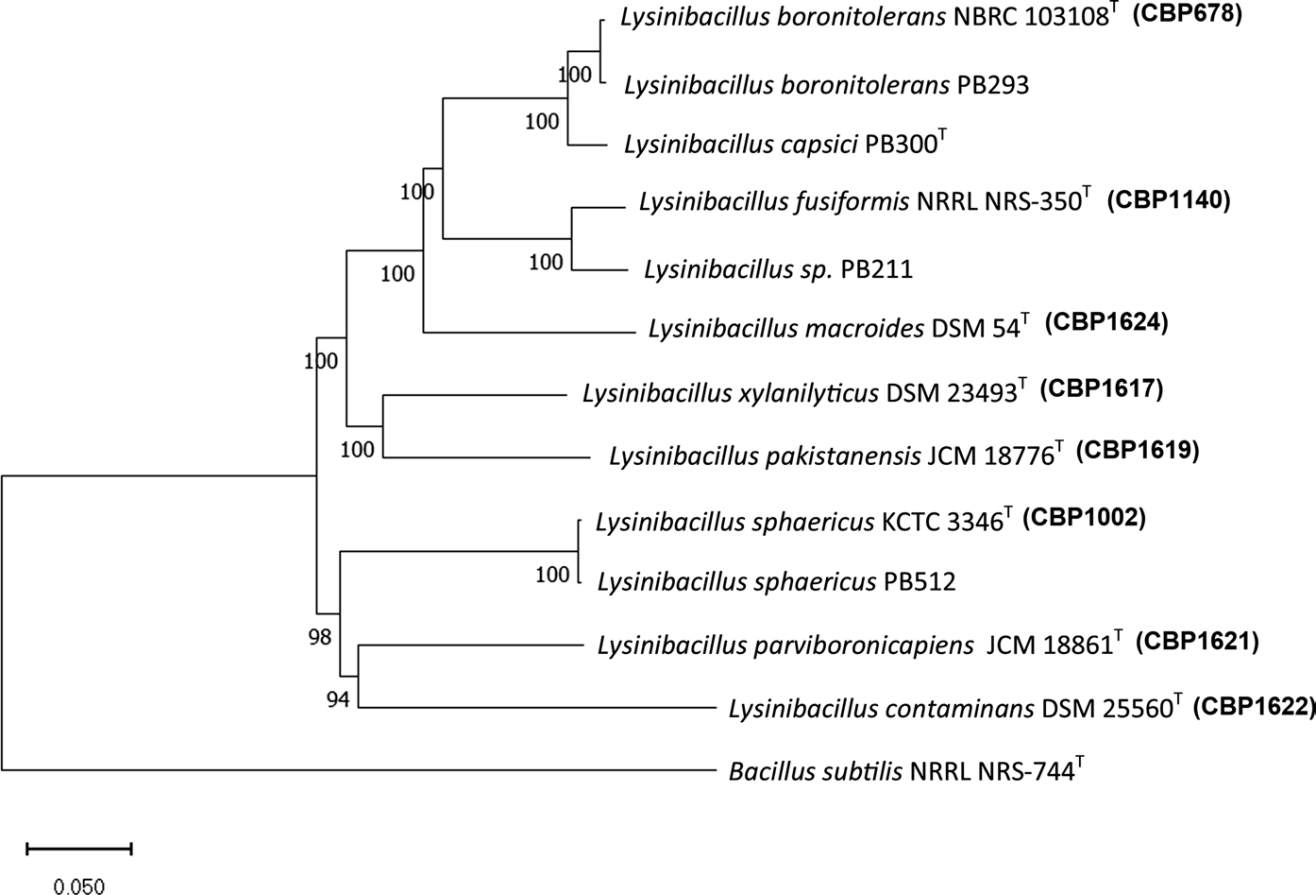
Phylogenetic Neighbor-joining tree reconstructed from the core genomes of selected strains of *Lysinibacillus* species (1185 genes). Bootstrap values > 50%, based on 1500 pseudoreplicates are indicated on branch points. *Bacillus subtilis* was used as an outgroup, only the relevant part of the tree is presented. The scale bar corresponds to 0.05 nucleotide substitutions per site

According to phylogenomic analysis, all studied strains belong to a monophyletic group identified as *Lysinibacillus spp* (Fig. 1). The taxonomy of the environmental strains that were bioprospected were determined. Strain PB512 was identified as a *Lysinibacillus sphaericus*. Strain PB293 was identified as a *Lysinibacillus boronitolerans*. Strain PB211 was identified as an undescribed new species of *Lysinibacillus,* because the average nucleotide identity between it and the closest type strain was less than 95%. Strain PB300 was recently described as a new species, *Lysinibacillus capsici* (Burkett-Cadena et al. 2019)*.*

*Lysinibacillus* is a remarkably diverse genus (Nakamura 2000; Hu et al. 2008; Berry 2012; Aimé et al. 2019). Currently, 37 species have been described (LPSN 2022), all of them share some phenotypic characteristics—such as their inability to metabolize carbohydrates, except for N-acetyl glucosamine—and its content of lysine and aspartic acid in the cell wall instead of diaminopimelic acid (Allievi et al. 2014; Gómez-Garzón et al. 2017). Although genomic evidence indicates this genus has a common ancestor well differentiated from other genera of the Bacilliaceae family, the low intra-species hybridization rate generates heterogeneous clades. Many species that have not yet been described are hypothesized (Xu et al. 2015; Gómez-Garzón et al. 2017; Clavados et al. 2017).

### Evaluation of *Lysinibacillus spp*. PGP activity on corn

The PB211, PB293, PB300, PB512, CBP1622, and CBP1624 strains presented PGPR activity in at least one of the evaluated variables (Table 1). The PGP effect was observed in most of the cases at higher inoculum concentration (10^8^ CFU/mL), except for PB300 and CBP1622, which exhibited this effect in lower concentrations. These results agree with Jinal et al. (2021), who observed PGPR activity and amelioration of stress physiological response in plants by the inoculation of *Lysinibacillus spp*. strains to a 10^8^ CFU/mL concentration. The number of lateral roots variable also follows this pattern. It was significantly stimulated from 10^4^ CFU/mL.

**Table 1:**
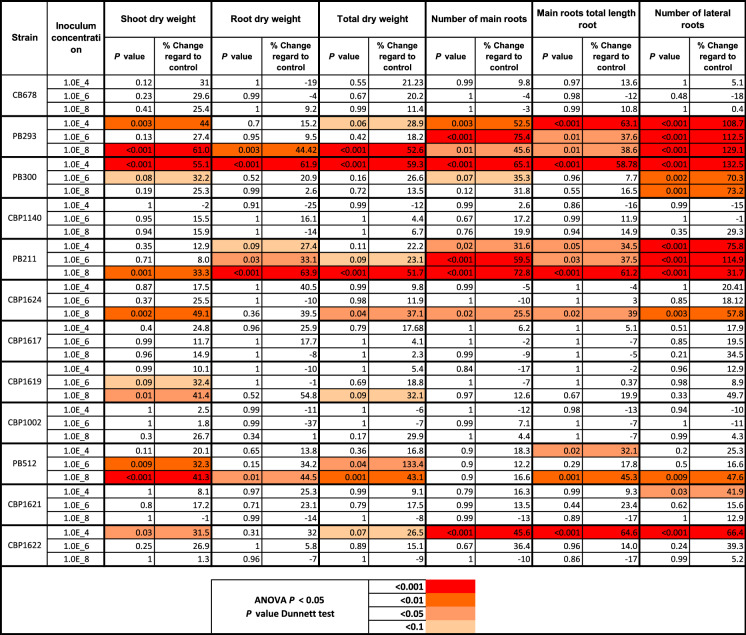
Effects of inoculating *Lysinibacillus spp*. strains on *Zea mays* ICA-109

The data were analyzed using the ANOVA test (*P* < 0.05). *P* values of the treatment's mean comparison with their respective control were calculated using Dunnett's test. Significative differences are labeled in color according to the scale in the bottom table. The mean percentage change of each treatment with its control also is presented. The box plots with the data distribution can be seen in Supplementary Material 1

Strains with PGP activity had a marked effect on root architecture variables, especially on lateral root proliferation (Table 1). This effect has been previously described with the addition of exogenous auxins (Alarcón et al. 2019; Jing and Strader 2019), IAA-producing bacteria (Spaepen et al. 2014; Batista et al. 2021), and other substances with auxin activity (Nardi et al. 2021). Some previous reports described the ability of *Lysisnibacillus sp*. strains to produce a high amount of IAA. They correlated this trait with its PGP effect (Naureen et al. 2017; Hanh and Mongkolthanaruk 2017).

*Lysinibacillus* is a relatively new genus. Ahmed et al. (2007) proposed the reclassification of two *Bacillus* species to *Lysinibacillus* since reports of its PGP potential are not abundant, even before being reclassified*. Lysinibacillus sphaericus* is widely known for its insecticidal activity due to the production of parasporal bodies. However, in recent years, research in PGP activity have raised considerably (Tiwari et al. 2019) showing interest of its biotechnological potential in agriculture (Ahsan and Shimizu 2021; Passera et al. 2021).

Sharma and Saharan (2015), Rahmoune et al. (2017), Gusain et al. (2017), Castellano-Hinojosa et al. (2018), and Jinal et al. (2021) are some of the authors who reported empirical evidence of *Lysinibacillus sp*. PGP-activity recently. In all cases, plant experiments were performed, and in vitro PGP traits were evaluated. However, causality evidence between these traits and results in plants was not presented. Strains belonging to the *Lysinibacillus sp*. genus tend to be better IAA producers than other genera of the Bacilleaceae family (Kim and Song 2012; Naureen et al. 2017; Pal and Sengupta 2019).

### Lysinibacillus spp. IAA bacterial production

**Fig. 2:**
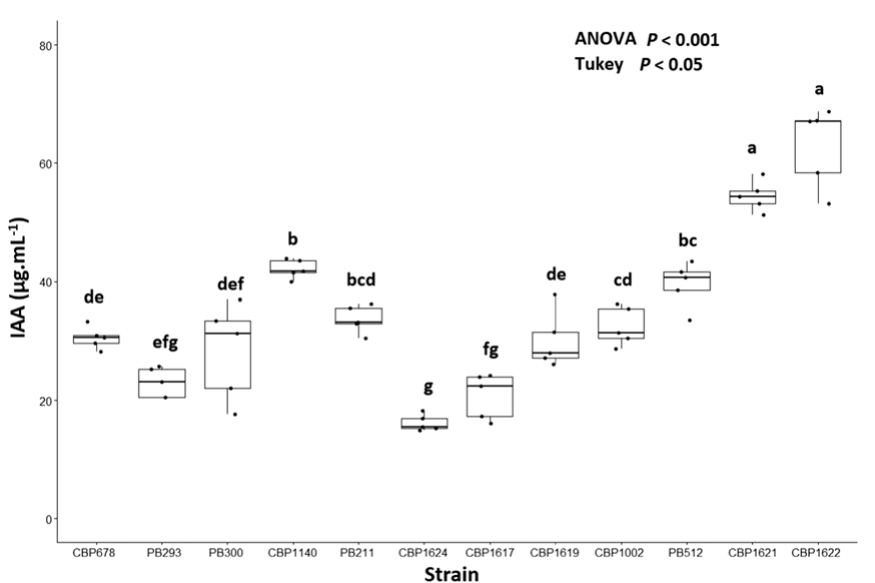
IAA production by strains of *Lysinibacillus spp*. Spectrophotometric detection was made with Salkowsky reagent, using a standard calibration curve with IAA known concentrations. The data was analyzed using the ANOVA (*P* < 0.05) test, and the comparison of means was calculated with Tukey's test (*P* < 0.05)

The IAA bacterial production was evaluated by spectrophotometry using the Salkowski reagent (Fig. 2). This is a sensitive method for IAA detection on bacterial culture broth supernatant. Of the possible compounds predicted for IAA pathways from the strain's genetic analysis (Table 2), only the Salkowski reagent generated a pink color reaction (detectable at 530–535 nm) with IAA and indole pyruvic acid (Gilbert et al. 2018). Indole pyruvic acid is a precursor without auxin activity (Kuźniar et al. 2021); it is not relevant for its PGP effect in plants. Studies with thin layer chromatography (TLC) and high-performance liquid chromatography (HPLC) indicated extra cell indole pyruvic acid production by aerobic endospore-forming bacteria (AEFB) is not significant compared to IAA production (Mohite 2013; Lee and Whang 2016). In other works, ipdC mutant bacterial strains (attenuated IAA production, but normal production of Indole Pyruvic Acid) the Indole detection by Salkowski was not significant (Ryu and Patten 2008).

**Table 2:**
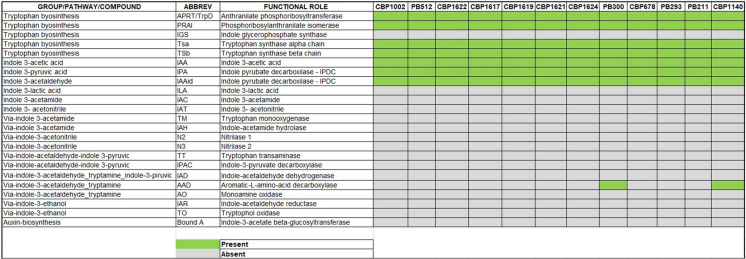
List of genes related to auxin synthesis found in *Lysinibacillus spp*

Genes functional role and prediction of indole compounds production pathway based on genome analysis. The detailed genes detected list and their sequences for the predicted products or compounds in each strain can be seen in Supplementary Material 2

IAA production displayed a high variation in the strains—between 20 and 70 μg/mL, (Fig. 2)—which has been previously described for this trait (Mohite 2013; Pal et al. 2017; Pal and Sengupta 2019). All strains used in this work presented the ability to produce IAA. However, not all of them expressed PGP activity, neither a similar pattern between IAA production nor PGP effect was observed. Although bacterial IAA production has been considered an important trait in promoting plant growth by bacteria (Barazani and Friedman 1999), their presence in the rhizosphere does not guarantee PGP activity (Nehl et al. 1997). The IAA bacterial production can also be a virulence factor in plant pathogenic bacteria (Chalupowicz et al. 2006; Kremer 2007). Furthermore, IAA bacterial production is usually associated with other PGP mechanisms, indicating the IAA production does not always have a direct effect (Ramírez and Kloepper 2010).

Most strains that showed PGP activity produced IAA from 20–50 μg mL, except for CBP1622, which was the highest producer and promoted corn growth at 10^4^. Strains that produce high amounts of IAA usually express their PGPR activity at low inoculum concentrations because variations in the inoculum concentration emulate the “dose effect” of auxins (Suarez et al. 2014; Mangmang et al. 2015; Kudoyarova et al. 2019).

CBP1621 and CBP1622, the highest IAA producers, were grouped in a separate clade in the phylogenomic tree, while CBP1624, the lowest IAA producer, was located separately in another clade; there is a concordance between the pattern of IAA bacterial production by *Lysinibacillus spp.* strains and the clades grouping in the phylogenomic tree. Therefore, this trait could be associated with the evolutionary inferences derived from the phylogenomic analysis. The co-evolutionary relationship of this trait in bacteria and plants has been previously described by Yue et al. (2014), and Blouin (2018).

### Bioinformatic identification of predicted genes associated with *Lysinibacillus spp*. IAA production

Genes associated with the synthesis of amino acid transferases, indole pyruvate decarboxylase (*ipdC*), and aldehyde dehydrogenases (*aldH*) were detected in all strains. It is possible to infer these strains use the indole pyruvic acid pathway (IPA) to synthesize IAA (Table 2), which is highly frequent in *Firmicutes* and dominant in *Bacilli* (Shao et al. 2015; Zhang et al. 2019; Alviar et al. 2021). Additionally, a gene for the enzyme aromatic-L-aminoacid decarboxylase was detected in PB300 and CBP1140. This enzyme is linked to the tryptamine pathway (TPM). Table 2 describes the group of genes, pathways, and compounds predicted for each strain as well as the functional role assigned.

The coexistence of two or more IAA bacterial synthesis pathways in rhizobacteria is a plausible phenomenon (Kim and Song 2012; Zhang et al. 2019). Batista et al. (2021) described the presence of IPA and TPM pathways in a *Bacillus thuringiensis* strain. However, the co-occurrence of more than one pathway does not increase the IAA production, as seen in the PB300 and CBP1140 results. The mechanisms that regulate IAA synthesis and its overproduction in bacteria are not yet well known (Zhang et al. 2019). Identifying these pathways help us to understand the *Lysinibacillus spp*. IAA-production role on plant growth. The IPA and TPM pathways are typical in PGPRs unlike plant-pathogenic bacteria, whose predominant pathway for IAA synthesis is Indole Acetamide (IAM) (Patten et al. 2013). For example, *Pseudomonas syringae* pv. savastanoi and *P. agglomerans* pv. gypsophiliae use IAM as a virulence mechanism for gall formation (Patten et al. 2013; Kunkel and Harper 2018).

Genes for synthesis of anthranilate phosphoribosyltransferase, phosphoribosyl-anthranilate isomerase, tryptophan synthase alpha chain, and tryptophan synthase alpha chain were detected in all strains. These enzymes are associated with tryptophan synthesis and metabolism. Some variations in these genes were linked to their individual strains or subsets (Supplementary Material 2). These variations could explain the differences in IAA production in vitro (Patten et al. 2013).

A gene for amino-L-phenylalanine methyltransferase (Phe-ATT) was found only in PB512 and CBP1002. This coincides with the phylogenomic analysis, where these two strains belong to the same species, *L. sphaericus* (Fig. 1). Aminotransferases (ATTs) participate in the oxidative catalysis of aromatic amino acids (Pedraza et al. 2004; Alkhalaf and Ryan 2015), which are necessary for the conversion of tryptophan to indole-3-pyruvate during IAA synthesis via IPA pathway (Alkhalaf and Ryan 2015; Spaepen and Vanderleyden 2011). Phenylalanine increases the bacterial enzyme activity of indole-pyruvate decarboxylase, like tryptophan (Molina et al. 2018). Bacteria metabolize phenylalanine through the IPA pathway to produce phenylacetic acid (PAA), not IAA (Ryu and Patten 2008; Wang et al 2016). PAA is a compound with auxin-like activity (Somers et al. 2005; Wang et al. 2016). Tryptophan-independent pathways for IAA synthesis have not yet been well characterized (Keswani et al. 2020). Therefore, future trials should evaluate IAA and PAA production by PB512 and CBP1002 using tryptophan and phenylalanine as precursors. The presence of Phe-ATT in these two strains could be relevant to explain their PGPR potential.

### Auxin-like activity of bacterial filtrates: coleoptile elongation test

**Fig. 3:**
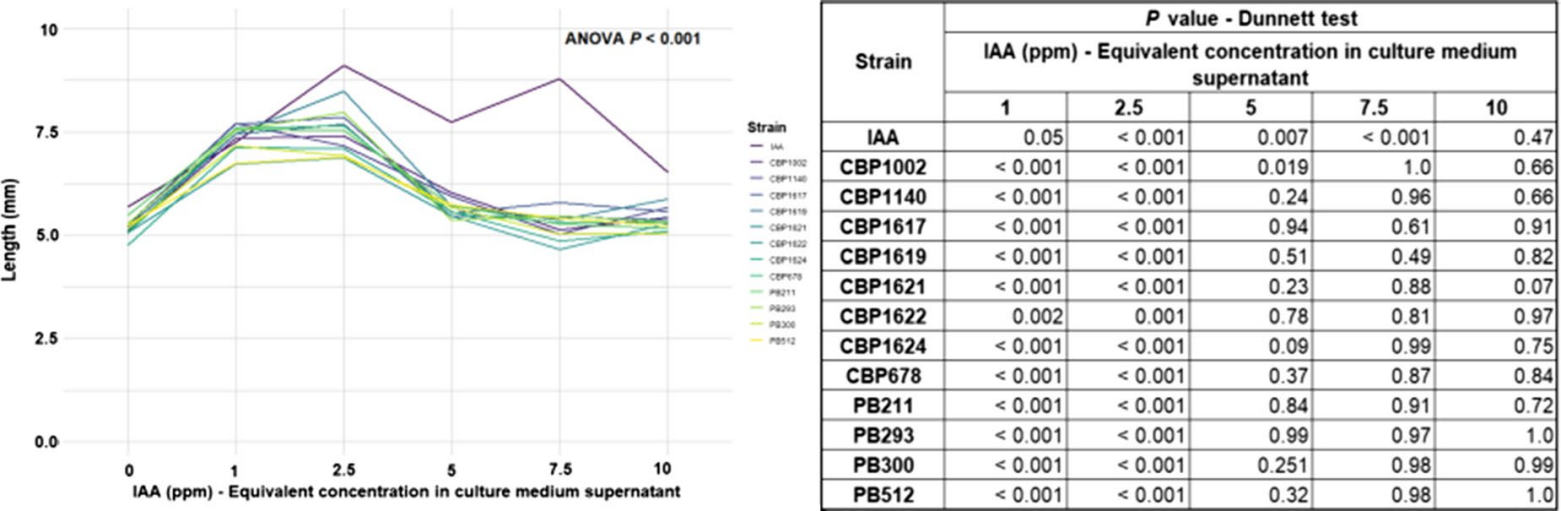
Coleoptiles elongation by supernatant filtrates of *Lysinibacillus spp*. liquid culture. The results are expressed in coleoptile length (mm), the colored lines represent the elongation trends of the IAA equivalent concentration in the strains' supernatant. The purple line corresponds to known IAA concentrations. The data were analyzed using the ANOVA, and the filtrates of each strain were compared with their respective control using Dunnett's test. The P values generated by the comparison of the means can be seen in the attached table. The data distribution can be observed in supplementary material 4

The coleoptile elongation test results show the auxin effect of several IAA concentrations on corn coleoptiles. The highest elongation observed was 2.5 mg/L, but there were effects from 1–7.5 mg/L (ppm) (Fig. 3). These results agree with Wilson (1977), who standardized a coleoptile elongation test assay. He found the highest coleoptiles elongation in a similar range of concentrations. Coleoptiles are cylindrical organs that envelop the plumule of grass seedlings (Srivastava 2002; Nielsen 2010). This test consists of excising the coleoptile meristematic zone to eliminate the auxin sources.Segments of known length are submerged in a liquid suspension. If any substance in the medium has auxin activity, it will be possible to observe increases in the segment length and/or curvature (Thimann and Bonner 1933; Went 1935; Park et al. 2001).

In this way, extracellular filtrates obtained from IAA-producing bacterial cultures should increase the coleoptile length in an IAA similar concentration pattern (Idris et al. 2004). The results of the coleoptile elongation test show the extracellular filtrates of all *Lysinibacillus* strains had auxin-like activity with a similar elongation pattern among them (Fig. 3). Filtrates of all strains between 1–2.5 mg/L showed longer coleoptiles, which is reasonable because all filtrates were prepared to the same concentration range. The fact all *Lysinibacillus* spp. filtrates responded similarly to the same IAA concentrations is indirect evidence of the IAA-bacterial effect on the growth of plant tissue (Idris et al. 2004).

In a parallel experiment, the auxin-like effect of *Lysinibacillus spp*. bacterial suspensions at different inoculum concentrations on corn coleoptiles were evaluated (Supplementary material 3). All bacterial suspensions except for CBP1622, promoted coleoptile elongation in at least one of the inoculum concentrations. This agrees with Djordjevic et al. (2017) findings, who described the auxin-like activity in oat coleoptiles suspended directly in bacterial cultures. The cause of the non-auxin-like activity of the CBP1622 bacterial suspension is unknown. However, it could be related to the growth rate of the strain. In previous observations, slower growth of CBP1622 was detected compared to the other strains (data not shown). Although there wasn’t any observable correlation among the inoculum concentrations, the bacterial production of auxins, or the elongation of the coleoptiles, we were able to find evidence of auxin-like activity when directly inoculating *Lysinibacillus spp*. on plant tissue.

### In vitro experiments with wild and mutant plants: *Arabidopsis thaliana*

Mutants *A. thaliana* aux1-7/axr4-2 were used because they have some alterations in auxin signaling, this feature is important to determine the role of the IAA microbial production as a PGP trait (Contreras-Cornejo et al 2009). These double mutants have less IAA amount in their root system; therefore, they have a dwarf phenotype (Wilson et al. 1990; Hobbie and Estelle 1995; Swarup et al. 2004; Dharmasiri et al. 2006; Puga-Freitas et al. 2012). We hypothesized that the *Lysinibacillus spp.* inoculation near the root systems under in vitro conditions would display a reversion of the mutant phenotype.

**Fig. 4:**
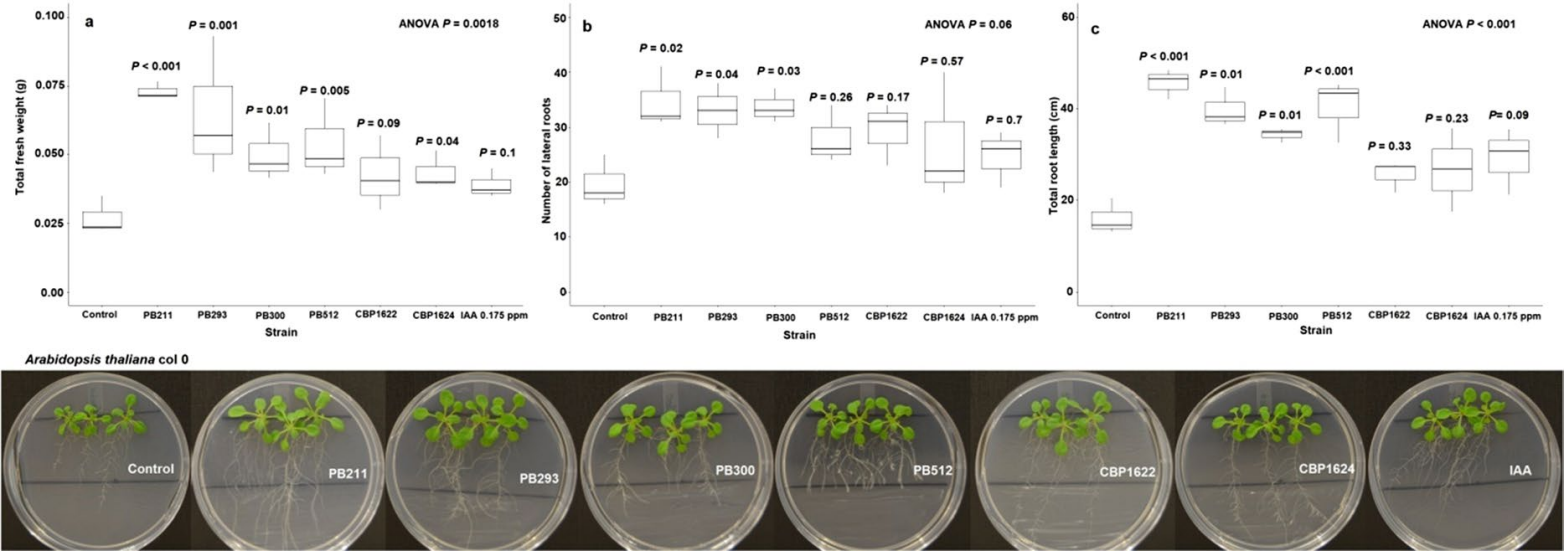
Effect of *Lysinibacillus spp*. inoculation on *Arabidopsis thaliana* col 0 (wild). Each box, represented by a letter, corresponds to a response variable. **a** Total fresh weight. **b** Number of lateral roots. **c** Total root length. The data were analyzed using ANOVA, and the means comparison with Dunnett's test was calculated. The treatments are labeled with their respective *P*-value. The bottom images correspond to representatives’ photos of each treatment

**Fig. 5:**
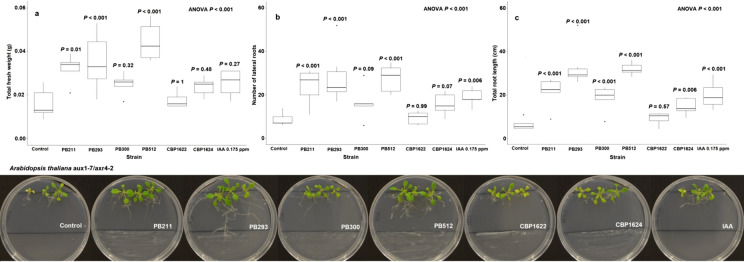
Effect of *Lysinibacillus spp*. inoculation on *Arabidopsis thaliana* aux1-7/axr4-2 (mutant). Each box, represented by a letter, corresponds to a response variable. **a** Total fresh weight. **b** Number of lateral roots. **c** Total root length. The data were analyzed using ANOVA, and the means comparison with Dunnett's test was calculated. The treatments are labeled with their respective* P*-value. The bottom images correspond to representatives’ photos of each treatment

PB211, PB293, PB300, PB512, and CBP1624 displayed PGP activity on *Arabidopsis thaliana* col 0 under in vitro conditions (Fig. 4). The inoculation of these strains caused also changes in fresh weight and root architecture of mutant *Arabidopsis thaliana* aux1-7/axr4-2 plants (Fig. 5), which indicates a partial reversion of the dwarf phenotype. This result agrees with several reports which indicated that the phenotype of aux1-7/axr4-2 is reverted with an auxin source that supplies the hormone lack in the root system, including IAA-producing microorganisms (Contreras-Cornejo et al. 2009; Puga-Freitas et al. 2012; Blouin 2018). Auxin signaling mutant plants to determine the role of the IAA microbial production as a PGP trait have been successfully used (Contreras-Cornejo et al. 2009; Batista et al. 2021).

The CBP1622 strain did not express PGP activity in wild *A. thaliana,* and it did not induce significant changes in the mutant plant's phenotype (Fig. 5). This result agrees with the absence of auxin-like activity of CBP1622 suspensions on corn coleoptiles (Supplement material 3). However, this strain exhibited PGPR activity on corn plants under greenhouse conditions and it is one of the higher IAA producers in this study. Although CBP1622 has PGPR activity in corn, it does not show this expression in other plant models. The PGP effect of some bacterial strains is the result of a co-evolutionary relationship. Therefore, root colonization could need a specificity level (Backer et al. 2018). Additionally, PGPR strains can have more than one action mechanism on plants. These can be interdependent to express PGP activity (Ramírez and Kloepper 2010; Duca et al. 2018). If CBP1622 presented PGP activity on corn and its cell suspensions did not present auxin-like activity in coleoptiles, the role of other PGP mechanisms on corn plants could be considered. Preliminary observations allow us to detect slower CBP1622 growth compared to the other strains (data not shown). Growth speed is an important variable in root colonization and for expressing PGPR activity (Beneduzi et al. 2012).

Other bacterial metabolites can also modify the root architecture, e.g., cytokinin, abscisic acid (ABA), volatile organic compounds (VOCs) (Grover et al. 2021), 1-aminocyclopropane-1-carboxylate (ACC), ethylene, and ACC deaminase enzyme (Zarei et al. 2020). However, some characteristics of these mutants’ points towards the auxin-like action. The axr4-2 mutant does not respond to other hormones. Only exogenous auxin addition has shown a consistent phenotypic response in these mutants (Hobbie and Estelle 1995; Li et al. 2006). In addition, the aux1-7 mutant is an allelic condition of AUX1, an auxin influx carrier epistatic to axr4 that prevents IAA translocation from leaf meristems to roots (Hobbie and Estelle 1995; Swarup et al. 2004), which leads this characteristic mutant phenotype. AXR4 mutation affects the AUX1 polar location causing an agravitropic phenotype by defective AUX1 trafficking in the root epidermis (Dharmasiri et al. 2006). This double mutation makes aux1-7/axr4-2 a suitable model for evaluating exogenous auxin activity (Nordström et al. 2004). The agravitropic damage by axr4 mutation causes a significant decrease in the number of lateral roots (Swarup et al. 2004). Therefore, the proliferation of lateral roots in this mutant only is explained by auxin absorption of an exogenous source (Swarup et al. 2004; Dharmasiri et al. 2006).

This root growth pattern was observed in the treatment of aux1-7/axr4-2 with IAA, PB211, PB293, PB300, PB512, and CBP1624 (Fig. 5). The changes observed in the mutant phenotype with the *Lysinibacillus spp*. inoculation could be explained, at least partially, by the IAA bacterial synthesis.

## Conclusion

In this work, the PGP activity of *Lysinibacillus spp.* was evaluated. We found a reasonable association between IAA-bacterial production and its PGP activity. *Lysinibacillus* is a bacterial genus with increasing agro-biotechnological interest; however, the available direct evidence on the role of plant-hormone production as a selection trait of strains is scarce (Poveda and González-Andrés 2021). Thus, the evidence presented in this study is a new contribution to the agro-biotechnological exploration of *Lysinibacillus* sp.

## Supplementary Information

Below is the link to the electronic supplementary material.

Supplementary file1 (PDF 1200 KB)

Supplementary file2 (XLSX 81 KB)

Supplementary file3 (PDF 804 KB)

Supplementary file4 (PDF 337 KB)

## Data Availability

The datasets used during the current manuscript are available from the corresponding author on reasonable request.
