# Supplementary figures and images for: Lysinibacillus spp.: an IAA-producing endospore forming-bacteria that promotes plant growth
Source: Antonie Van Leeuwenhoek. 2023 May 3;116(7):615–30. doi: 10.1007/s10482-023-01828-x (PMC10257616; doi:10.1007/s10482-023-01828-x)

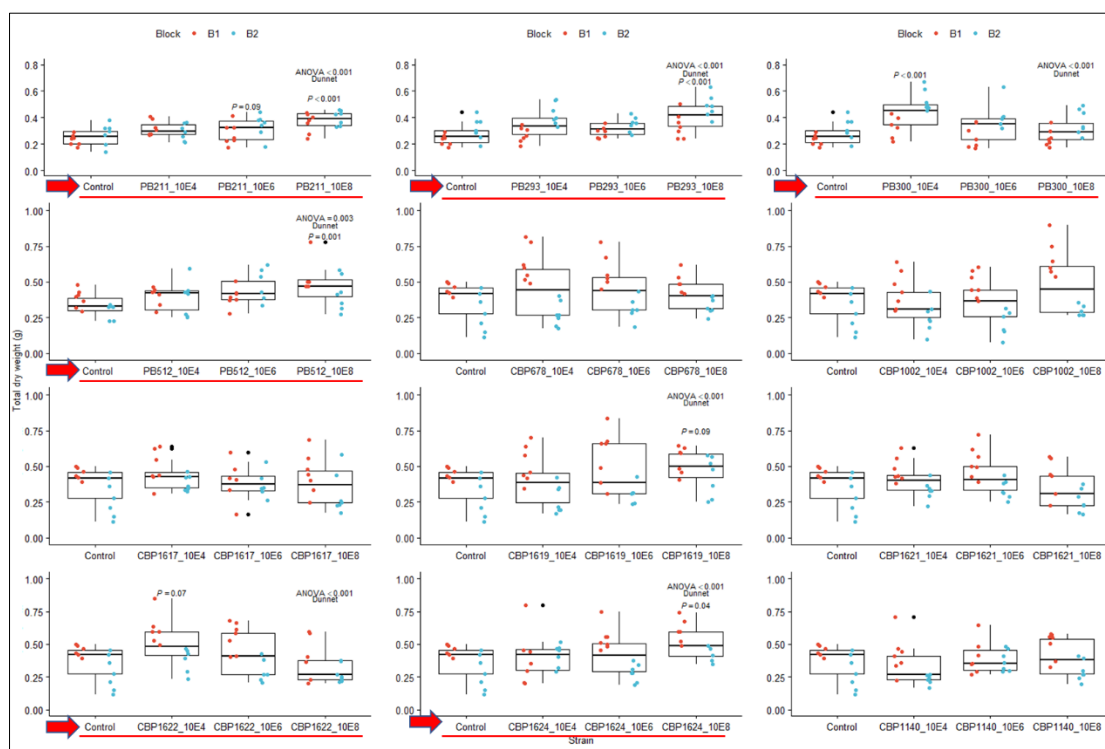

### Number of main roots

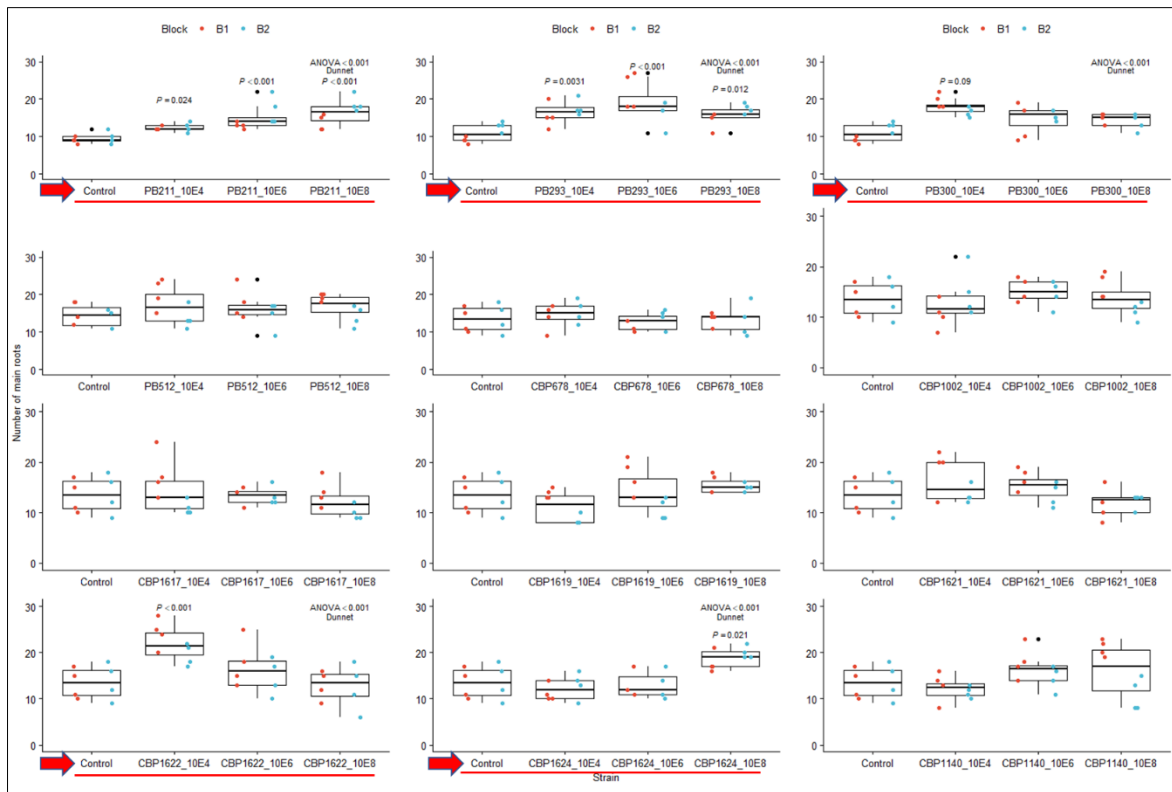

### Total root length

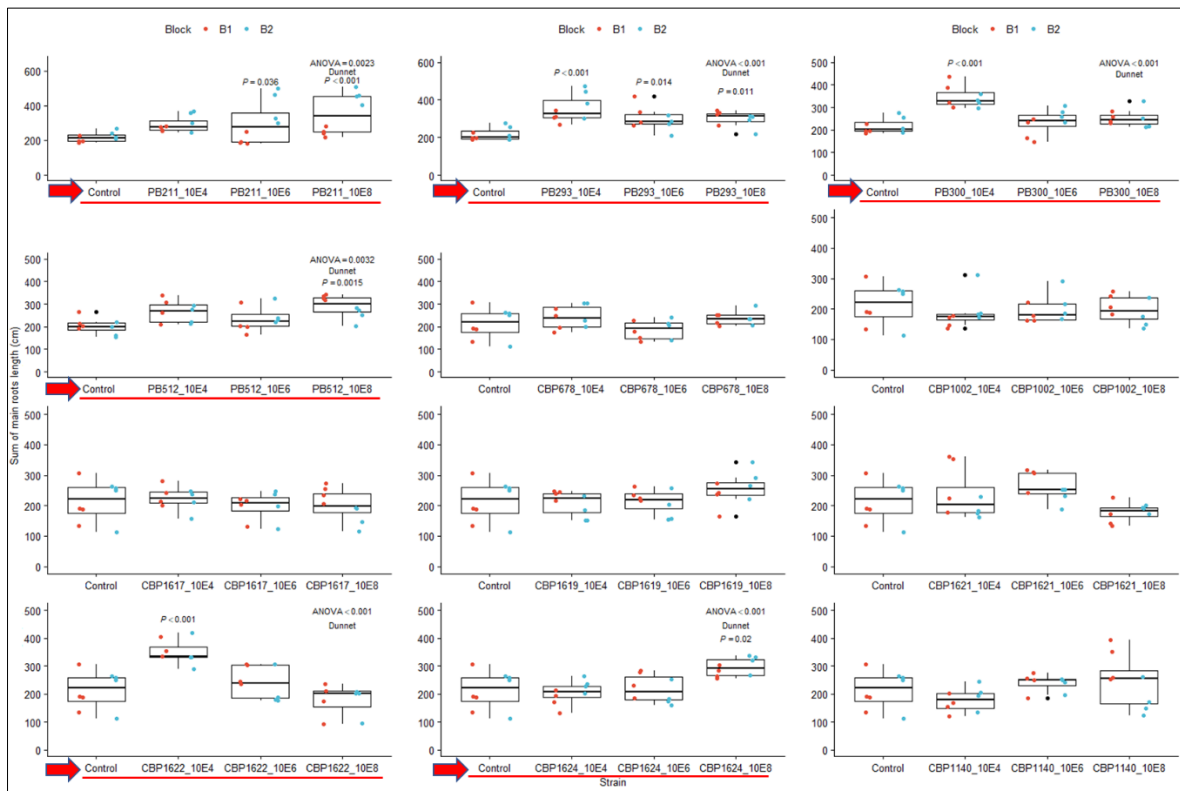

### Number of lateral roots

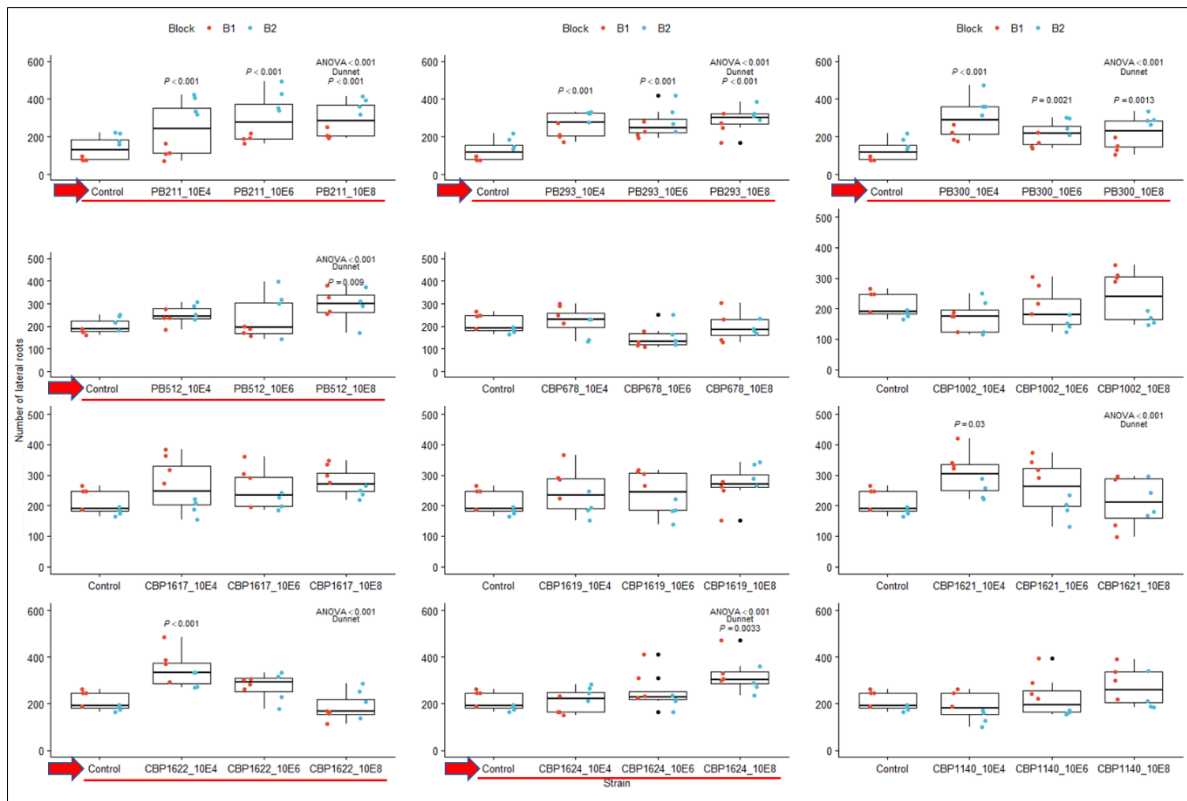

## Convex hull area

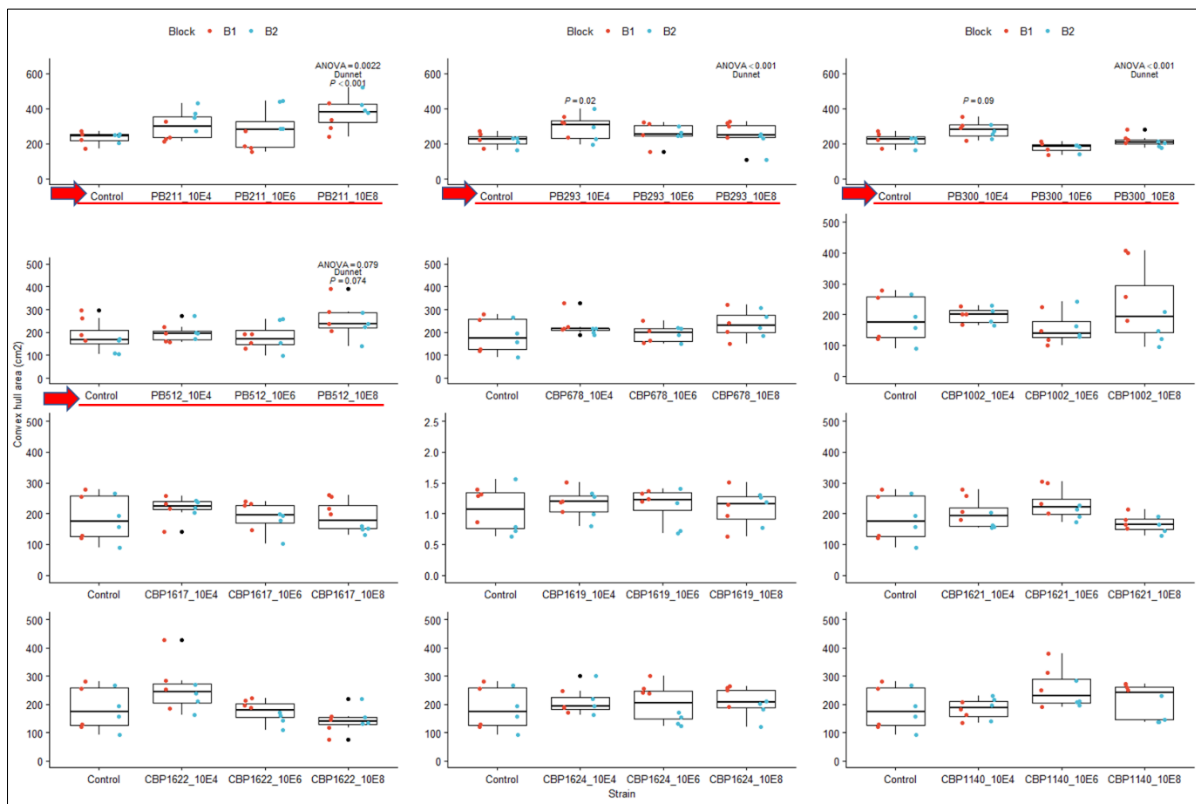

Supplement: Supplementary file 1 — Supplementary file1 (PDF 1200 KB) [file 10482_2023_1828_MOESM1_ESM.pdf]
