# Supplementary material for: Lysinibacillus spp.: an IAA-producing endospore forming-bacteria that promotes plant growth
Source: Antonie Van Leeuwenhoek. 2023 May 3;116(7):615–30. doi: 10.1007/s10482-023-01828-x (PMC10257616; doi:10.1007/s10482-023-01828-x)
Supplement: Supplementary file 3 — Supplementary file3 (PDF 804 KB) [file 10482_2023_1828_MOESM3_ESM.pdf]

Lysinibacillus spp.: An IAA-producing endospore forming-bacteria that promotes plant growth.

Journal: Antonie van Leeuwenhoek Journal of Microbiology

Manuel Pantoja-Guerra iD[1,2*], Marleny Burkett-Cadena[3], Johanna Cadena[3], Christopher A Dunlap iD[4] Camilo A. Ramírez[1]

[1]Universidad de Antioquia, Instituto de Biología, Medellín – Colombia

[2]Corporación Universitaria Lasallista, Caldas, Antioquia – Colombia

[3]Plant Response Biotech, Plant City, FL, USA

[4]United States Department of Agriculture, Agricultural Research Service, National Center for Agricultural Utilization Research, Crop Bioprotection Research Unit, 1815 N University, Peoria, IL, USA

*Corresponding author: manuel.pantojag@udea.edu.co

Supplementary material 3. Elongation coleoptile test: Trial bacterial suspensions

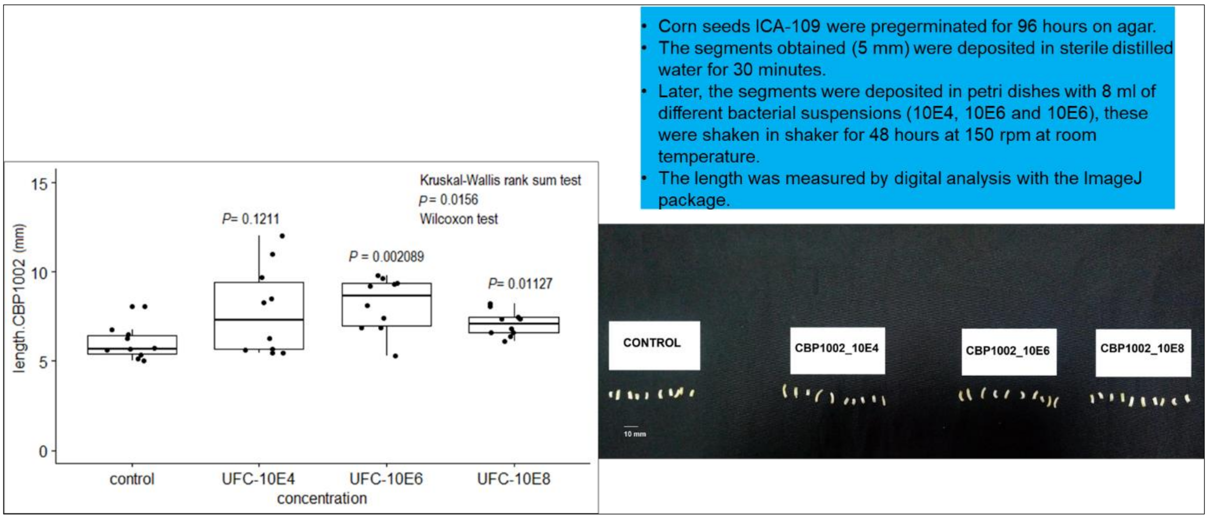

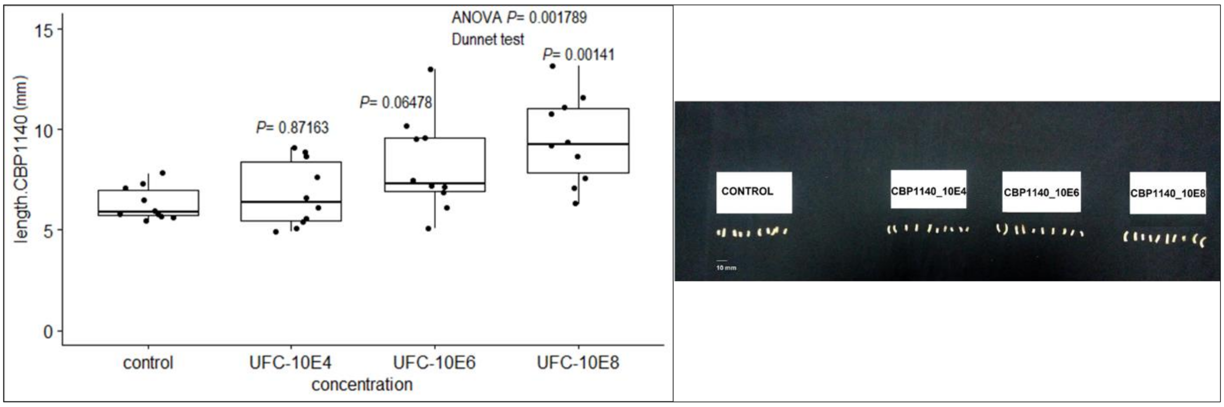

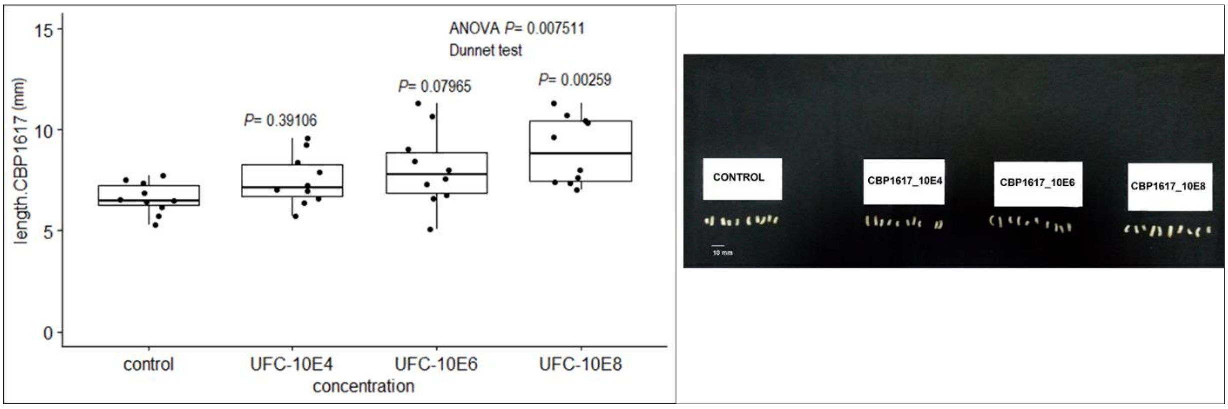
ANOVA P= 0.007511
Dunnet test
P= 0.39106
P= 0.07965
P= 0.00259
length.CBP1617 (mm)
control
UFC-10E4
UFC-10E6
UFC-10E8
concentration
CONTROL
CBP1617_10E4
CBP1617_10E6
CBP1617_10E8

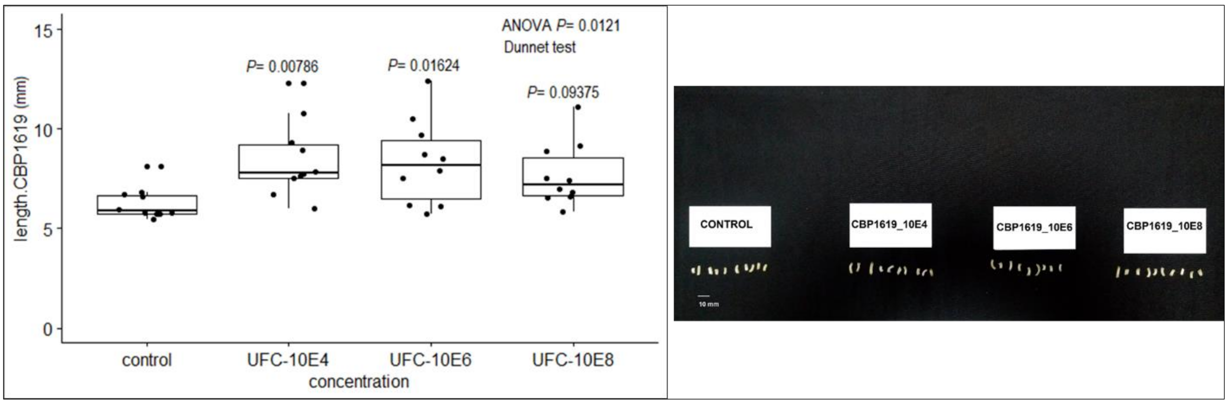
ANOVA P= 0.0121
Dunnet test
P= 0.00786
P= 0.01624
P= 0.09375
length.CBP1619 (mm)
control
UFC-10E4
UFC-10E6
UFC-10E8
concentration
CONTROL
CBP1619_10E4
CBP1619_10E6
CBP1619_10E8

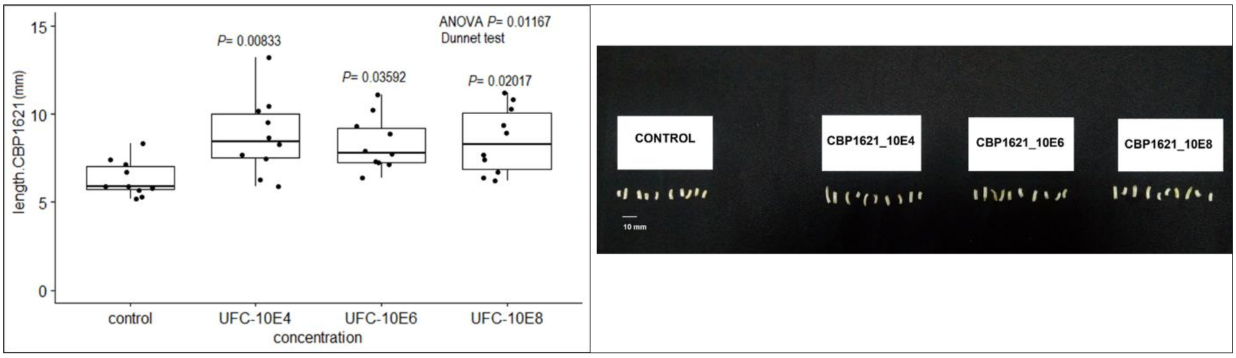
ANOVA P= 0.01167
Dunnet test
P= 0.00833
P= 0.03592
P= 0.02017
length.CBP1621 (mm)
control
UFC-10E4
UFC-10E6
UFC-10E8
concentration
CONTROL
CBP1621_10E4
CBP1621_10E6
CBP1621_10E8

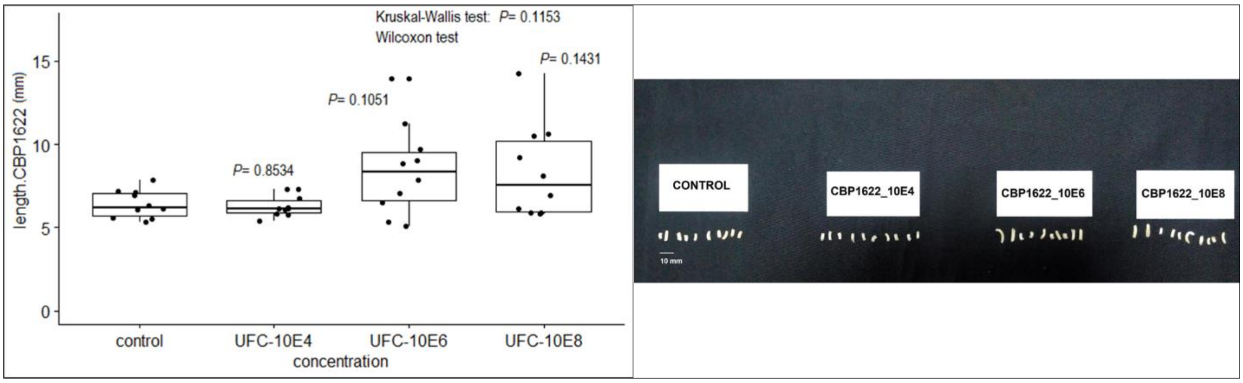
Kruskal-Wallis test: P= 0.1153
Wilcoxon test
P= 0.8534
P= 0.1051
P= 0.1431
length.CBP1622 (mm)
control
UFC-10E4
UFC-10E6
UFC-10E8
concentration
CONTROL
CBP1622_10E4
CBP1622_10E6
CBP1622_10E8

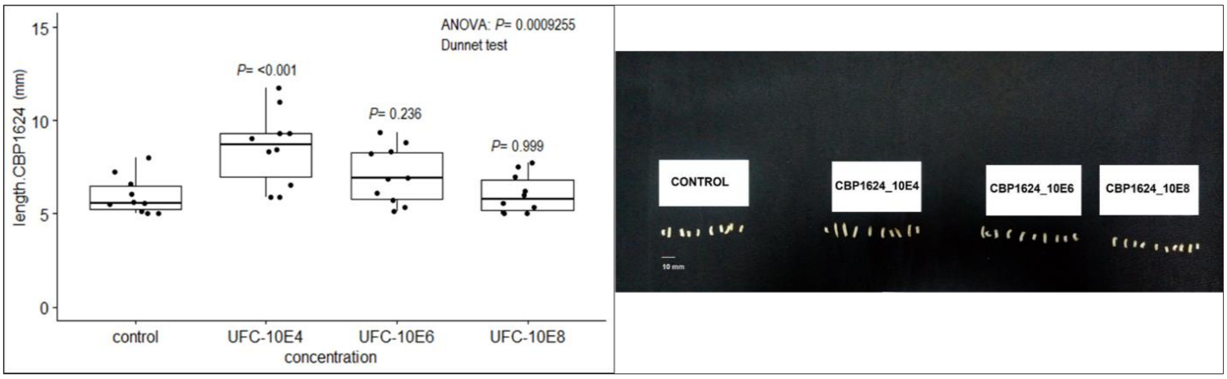
ANOVA: P= 0.0009255
Dunnet test
length.CBP1624 (mm)
P= <0.001
P= 0.236
P= 0.999
control
UFC-10E4
UFC-10E6
UFC-10E8
concentration
CONTROL
CBP1624_10E4
CBP1624_10E6
CBP1624_10E8
10 mm

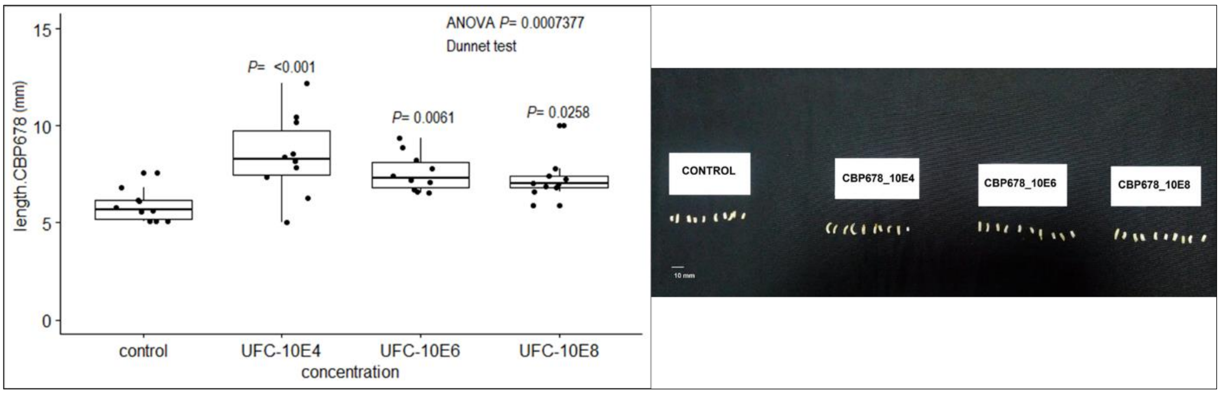
ANOVA P= 0.0007377
Dunnet test
length.CBP678 (mm)
P= <0.001
P= 0.0061
P= 0.0258
control
UFC-10E4
UFC-10E6
UFC-10E8
concentration
CONTROL
CBP678_10E4
CBP678_10E6
CBP678_10E8
10 mm

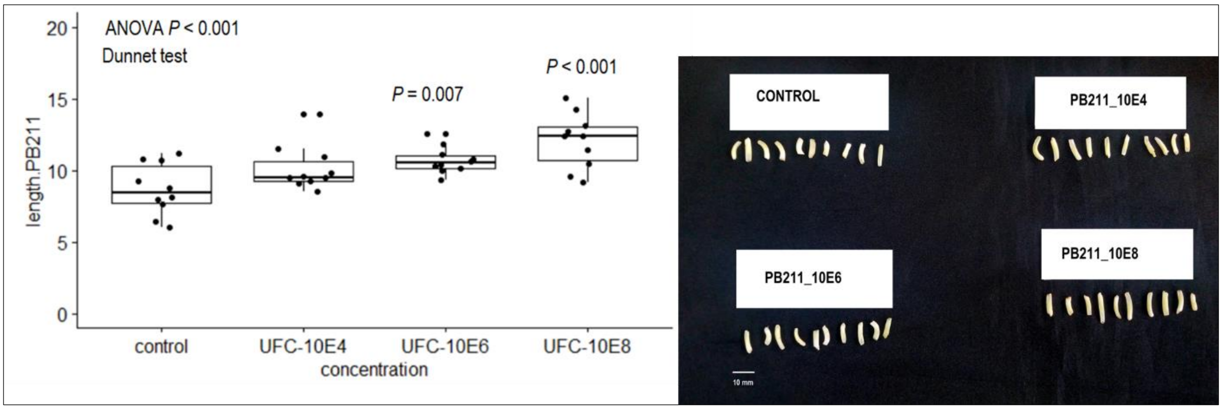
ANOVA P < 0.001
Dunnet test
length.PB211
P = 0.007
P < 0.001
control
UFC-10E4
UFC-10E6
UFC-10E8
concentration
CONTROL
PB211_10E4
PB211_10E6
PB211_10E8
10 mm

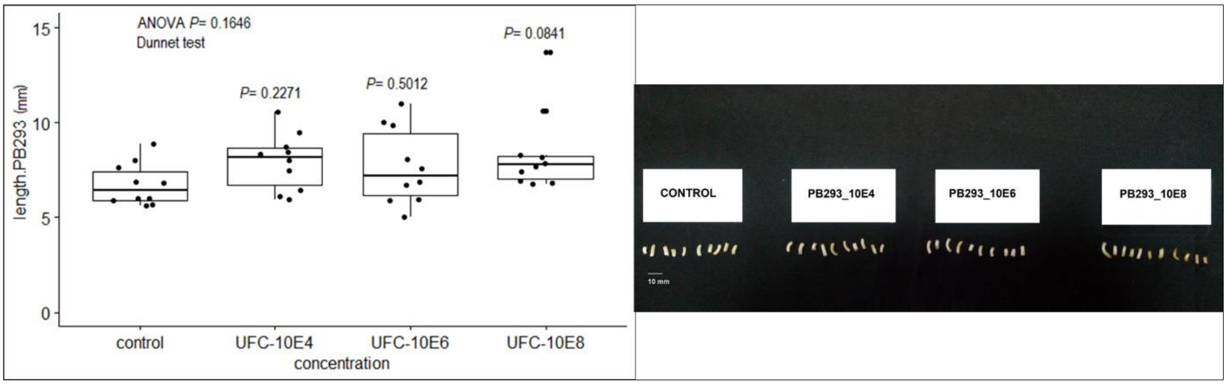
ANOVA P= 0.1646
Dunnet test
length.PB293 (mm)
P= 0.2271
P= 0.5012
P= 0.0841
control
UFC-10E4
UFC-10E6
UFC-10E8
concentration
CONTROL
PB293_10E4
PB293_10E6
PB293_10E8
10 mm

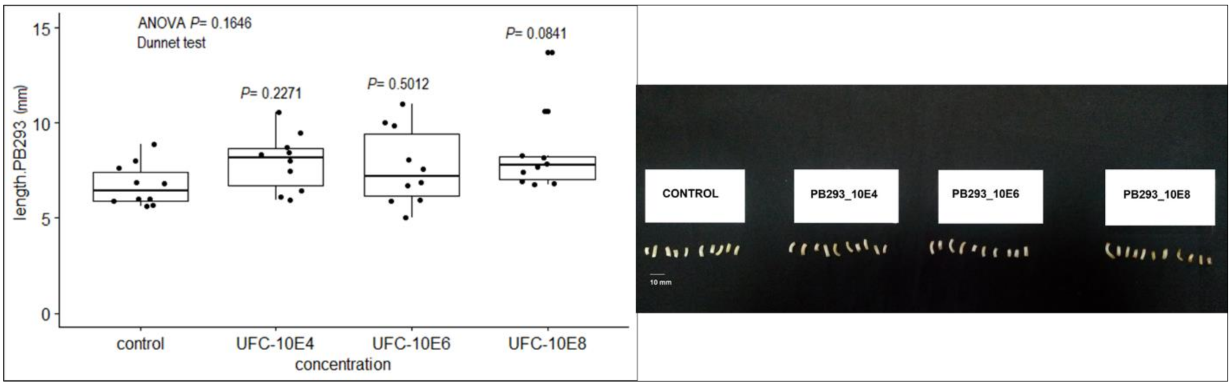
ANOVA P= 0.1646
Dunnet test
P= 0.2271
P= 0.5012
P= 0.0841
length.PB293 (mm)
15
10
5
0
control
UFC-10E4
UFC-10E6
UFC-10E8
concentration
CONTROL
PB293_10E4
PB293_10E6
PB293_10E8
10 mm

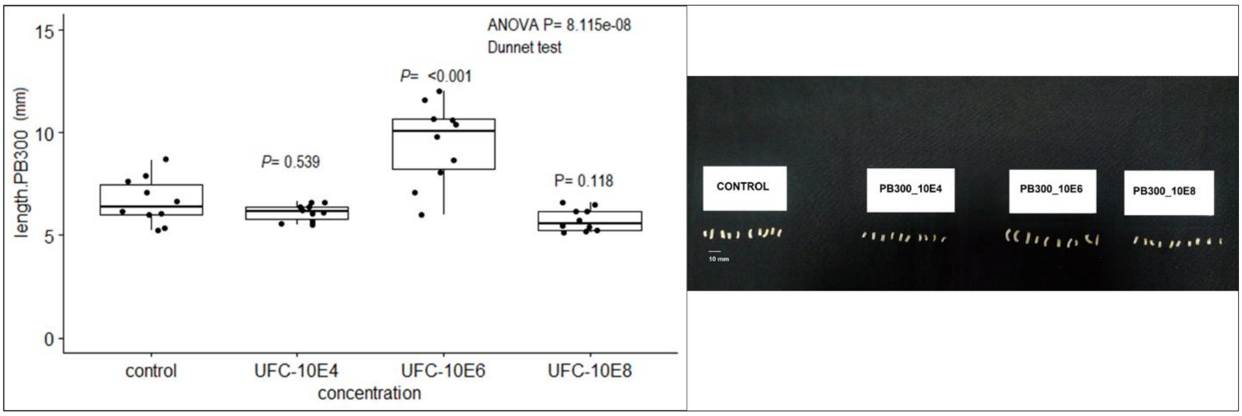
ANOVA P= 8.115e-08
Dunnet test
P= <0.001
P= 0.539
P= 0.118
length.PB300 (mm)
15
10
5
0
control
UFC-10E4
UFC-10E6
UFC-10E8
concentration
CONTROL
PB300_10E4
PB300_10E6
PB300_10E8
10 mm

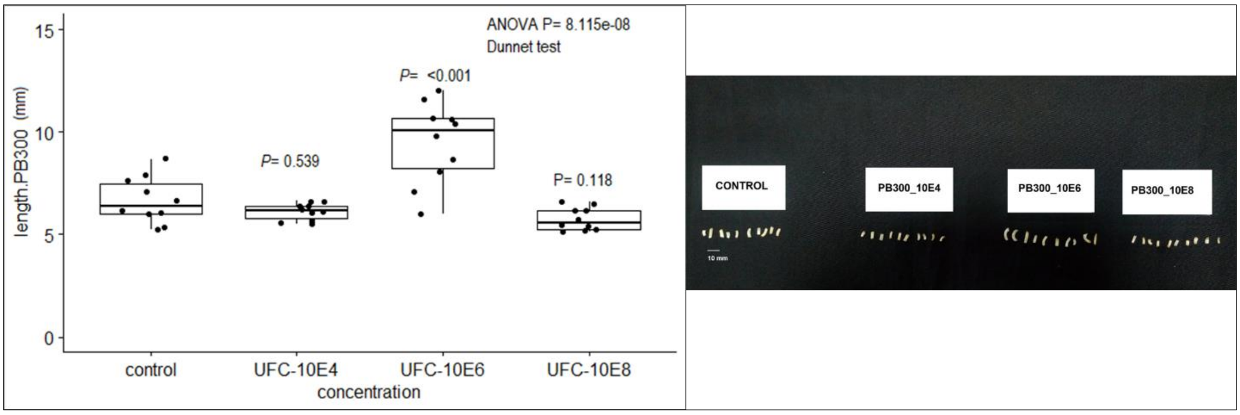
ANOVA P= 8.115e-08
Dunnet test
P= <0.001
P= 0.539
P= 0.118
length.PB300 (mm)
15
10
5
0
control
UFC-10E4
UFC-10E6
UFC-10E8
concentration
CONTROL
PB300_10E4
PB300_10E6
PB300_10E8
10 mm
