# Supplementary material for: Lysinibacillus spp.: an IAA-producing endospore forming-bacteria that promotes plant growth
Source: Antonie Van Leeuwenhoek. 2023 May 3;116(7):615–30. doi: 10.1007/s10482-023-01828-x (PMC10257616; doi:10.1007/s10482-023-01828-x)

Lysinibacillus spp.: An IAA-producing endospore forming-bacteria that promotes plant growth.

Journal: Antonie van Leeuwenhoek Journal of Microbiology

Manuel Pantoja-Guerra iD[1,2*], Marleny Burkett-Cadena[3], Johanna Cadena[3], Christopher A Dunlap iD[4] Camilo A. Ramírez[1]
[1]Universidad de Antioquia, Instituto de Biología, Medellín – Colombia
[2]Corporación Universitaria Lasallista, Caldas, Antioquia – Colombia
[3]Plant Response Biotech, Plant City, FL, USA
[4]United States Department of Agriculture, Agricultural Research Service, National Center for Agricultural Utilization Research, Crop Bioprotection Research Unit, 1815 N University, Peoria, IL, USA

[*]Corresponding author: manuel.pantojag@udea.edu.co

Supplementary material 4. Coleoptiles elongation by supernatant filtrates of *Lysinibacillus* spp. Box-plots with data distribution.

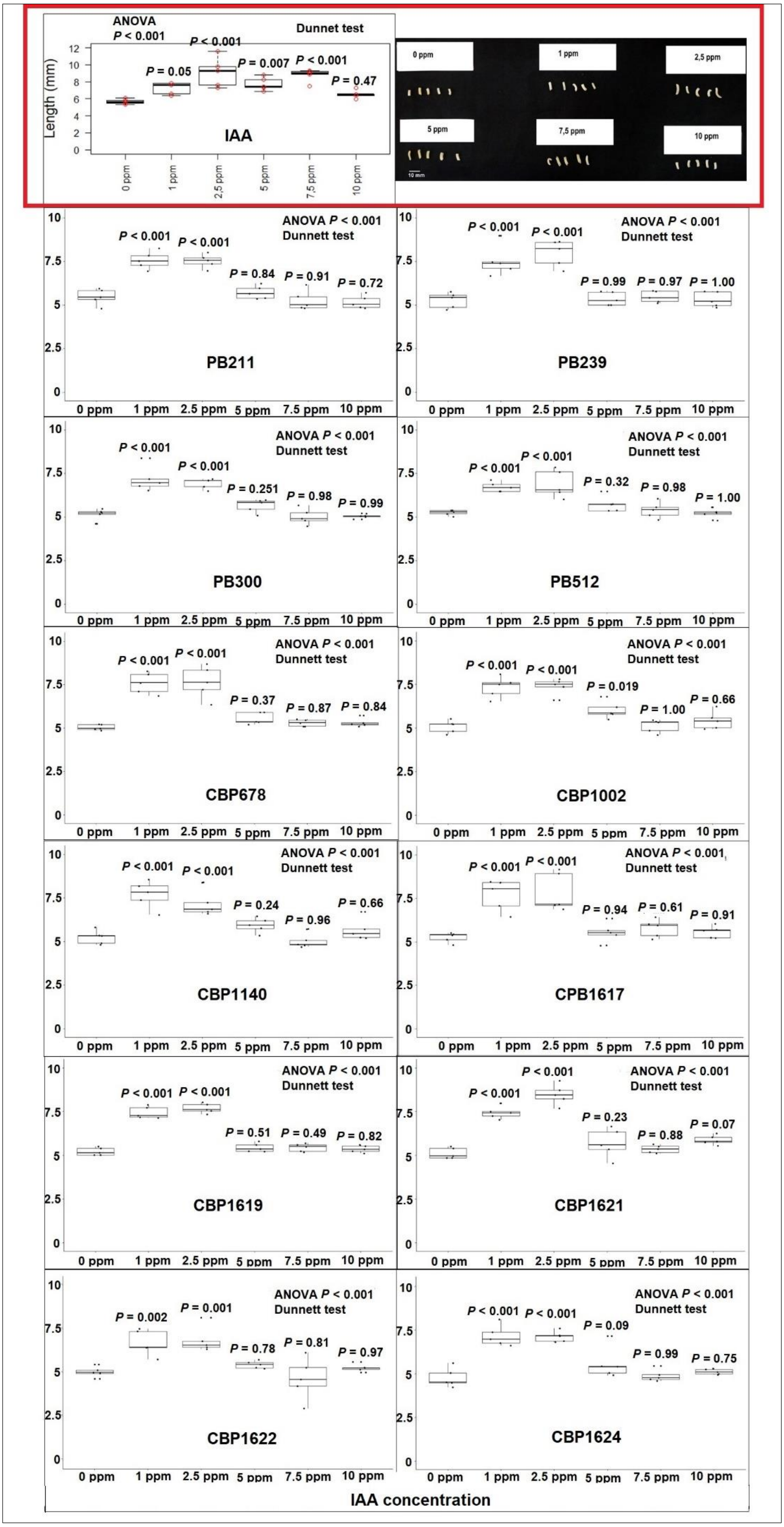

Supplement: Supplementary file 4 — Supplementary file4 (PDF 337 KB) [file 10482_2023_1828_MOESM4_ESM.pdf]
